# Upregulation of LRRC8A by m^5^C modification-mediated mRNA stability suppresses apoptosis and facilitates tumorigenesis in cervical cancer

**DOI:** 10.7150/ijbs.79205

**Published:** 2023-01-01

**Authors:** Yanjie Chen, Xinzhao Zuo, Qinglv Wei, Jie Xu, Xiaoyi Liu, Shiling Liu, Haocheng Wang, Qingya Luo, Yuya Wang, Yu Yang, Hongyan Zhao, Jing Xu, Tao Liu, Ping Yi

**Affiliations:** 1 Department of Obstetrics and Gynecology, The Third Affiliated Hospital of Chongqing Medical University, Chongqing 401120, China.; 2 Department of Obstetrics and Gynecology, Daping Hospital, Army Medical University, Chongqing 400042, China.; 3 Department of Pathology, Southwest Hospital, Army Medical Universtiy, Chongqing 400038, China.; 4 Institute of Basic Medical Sciences, Hubei University of Medicine, Shiyan, Hubei 442000, China.

## Abstract

Cervical cancer (CC) is one of the most common gynecological malignancies with poor prognosis for advanced CC patients. LRRC8A is a volume-regulated anion channel protein involved in cellular homeostasis, but its role in CC remains largely unknown. In this study, we found that LRRC8A is elevated in CC and associated with poor prognosis. LRRC8A maintains cell survivals under the hypotonic condition, and promotes tumorigenesis through apoptosis suppression *in vitro* and *in vivo*. Notably, LRRC8A is upregulated by NSUN2-mediated m^5^C modification. m^5^C modified-LRRC8A mRNA is bound by the RNA binding protein YBX1 followed by the increased RNA stability. Moreover, loss of NSUN2 suppresses the proliferation and metastasis of CC cells, and NSUN2 expression is positively correlated with LRRC8A expression in CC. Altogether, our study demonstrates that the NSUN2-m^5^C-LRRC8A axis is crucial and would be a potential therapeutic target for CC.

## Introduction

Cervical cancer (CC) is one of the most common gynecological malignancies throughout the world and has emerged as a prominent public health focus. According to the statistics in 2020, there are over 600,000 new cases and 340,000 deaths globally each year 1-3. Persistent infection of high-risk human papillomavirus (HPV) causes the majority of CC, thus making it a rare preventable cancer. It can be prevented through HPV vaccination and treatment with surgical therapy, chemotherapy, and radiotherapy. However, there is still a high proportion of advanced CC at initial diagnosis with 52% of cases with regional involvement or distant metastasis, resulting in the poor prognosis of patients with advanced CC 4. Therefore, CC remains a great medical challenge and requires new potential targets.

Maintenance of a relatively constant cell volume is the critical cell function to keep cellular homeostasis. Cell proliferation and apoptosis are associated with normotonic alterations of cell volume 5, 6. The variation of osmotic pressures is a crucial factor to result in cell volume regulation 7. Extracellular hyposmolality or intracellular hyperosmosis induces cell swelling, and osmotically swollen cells restore their original volume and protect themselves via a process called regulatory volume decrease (RVD) 8. LRRC8A was recently identified as an essential component of the volume-regulated anion channel (VRAC) which is activated when a cell swelling 9. VRAC plays an essential role in RVD process, and LRRC8A was the core functional component of VRAC 10. LRRC8A can promote the proliferation and migration of cancer cells in a variety of cancers 11. In hepatocellular carcinoma and gastric cancer, LRRC8A was highly expressed, and knockdown of LRRC8A significantly inhibited cell proliferation and migration, and induced apoptosis, suggesting that the constant cell volume might be involved in the malignancy of tumors 12, 13.

Recently, the association between abnormal RNA modifications and tumorigenesis has attracted widespread attention 14. The 5-methylcytosine (m^5^C) RNA modification, as an abundant modification, plays an important role in RNA metabolic processes, such as RNA stability, RNA processing, RNA export, and mRNA translation 15-20. Dysregulation of m^5^C modification has been linked to various diseases including cancer 21. In bladder cancer, m^5^C deposited in 3' UTR of heparin binding growth factor (HDGF) mRNA enhances its stability, and therefore exhibits the essential oncogenic role 16. LIN28B binds to m^5^C-modified GRB2 mRNA, promotes its expression, and ultimately activates PI3K/AKT and ERK/MAPK oncogenic signaling pathways in esophageal squamous cell carcinoma 17. m^5^C modification exists in 94% of mRNA in HeLa cells, indicating that the regulation of RNA fate by m^5^C modification may be an important mechanism leading to the occurrence and development of cervical cancer. However, whether or not the m^5^C modification affects tumorigenesis by regulating cell homeostasis is little known.

Our results revealed that LRRC8A mRNA is subjected to m^5^C modification and upregulated in CC cells. Higher expression of LRRC8A predicts poor prognosis for CC patients. Depletion of LRRC8A induces apoptosis under the hypotonic condition and suppresses tumorigenesis of CC cells. The m^5^C metyltansferasse NSUN2 mediates m^5^C modification of LRRC8A mRNA and increases its stability. Our study demonstrates that the NSUN2-m^5^C-LRRC8A axis is crucial for tumorigenesis of CC.

## Materials and methods

### Cervical tumor samples and cell culture

All cervical squamous cell carcinoma and normal cervical tissue specimens were obtained from patients undergoing surgery in Daping Hospital of the Army Military Medical University from 2020-03-01 to 2020-05-30. All subjects were informed the consent, and all these specimens were pathologically verified and reviewed by the institutional board of the Third Affiliated Hospital of Chongqing Medical University (2022033). HEK293T and HeLa cells were cultured in Dulbecco's Modified Eagle's medium (DMEM) high glucose medium (GIBCO, USA), and SiHa cells were cultured in minimum Eagle's medium (MEM) (GIBCO, USA). Medium was supplemented with 10% fetal bovine serum (FBS) (GIBCO, USA) and 1% penicillin-streptomycin (Solarbio, China), and cells were incubated at 37°C with 5% CO_2_ in a humidified atmosphere. Cells where indicated were treated with the AKT phosphorylation agonist SC79 (MedChemExpress, USA).

### Plasmids, cell transfection, and lentiviral infection

For LRRC8A and NSUN2 knockdown, all shRNAs including shCtrl (scramble), sh1 and sh2 were cloned into pLKO.1 backbone with puromycin selection. The related sequences of shRNAs were shown in the supplementary table**.** For LRRC8A overexpression, full-length LRR8CA coding sequence was cloned into pCDNA3.1 vector (Youbio). Overexpression of NSUN2 was conducted by the full-length NSUN2 coding sequence cloned into pCDH vector (Addgene). NSUN2-WT and NSUN2-MUT (C271, C321) expression plasmids were constructed by using the pEnter vector (Addgene), and the empty vector was used as the negative control.

Plasmid transfection was conducted by using jetPRIME transfection (Polyplus, France). After 24 hours or later, the levels of mRNA and protein were measured. Lentiviral vectors were transfected into HEK293T cells with packaging vectors psPAX2 (#12260, Addgene) and pMD2.G (#12259, Addgene) by using jetPRIME transfection (Polyplus, France). Infectious lentivirus particles were harvested at 48 hours after transfection.

### Quantitative reverse transcription-polymerase chain reaction (qRT-PCR)

Trizol reagent (Sigma, USA) was used to extract total RNA from HeLa cells and SiHa cells. For mRNA expression analysis, qRT-PCR was performed by using SYBR Green Master Mix (Vazyme) on QuantStudioDx instrument (Life Technologies). A typical cycling condition included 95°C for 5 min followed by 40 cycles at 95°C for 10 s, 60°C for 30 s. GAPDH was used as the internal standard control. Each sample runs in triplicate. Primers used in qRT-PCR were listed in the supplementary table.

### Western blot

HeLa and SiHa cells were treated with lysis buffer containing protease inhibitors (APExBIO, USA) for western blot and IP (Beyotime, China). Cells were lysed on ice for 30 min, and the lysate was obtained by centrifugation at 12,000 rpm for 15 min. The concentration of proteins was measured by using a bicinchonininc acid BCA protein assay kit (Solarbio, China). Then the proteins were resolved by 10% SDS-PAGE and transferred on PVDF membranes (Millipore, USA). The membrane was blocked with 5% non-fat milk in TBS/Tween-20 for 1 hour at room temperature. After blocking, the membranes were blotted with primary antibodies against LRRC8A (24979S, CST, 1:1000), NSUN2 (20854-1-AP, Proteintech, 1:2000), Flag-tag (20543-1-AP, Proteintech, 1:2000), YBX1 (7944S, CST, 1:1000), Caspase-3 (9662S, CST, 1:1000), p-AKT (4060S, CST, 1;1000), AKT (60203-2-Ig, Proteintech, 1:1000), p-PI3K (CY6428. 1:500, Abways), PI3K (#4249, 1:1000, CST), or GAPDH (60004-1-Ig, Proteintech, 1:1000) overnight at 4°C. After being washed with TBS/Tween-20, the PVDF membranes were incubated with chemiluminescent secondary antibody (SA00001-1/SA00001-2, Proteintech, 1:10000).

### Immunohistochemistry (IHC)

After dewaxing the paraffin sections of tissues, heat induced antigen retrieval was conducted. The slides were blocked with goat serum (ZSGB-BIO, ZLI-9021) at 37°C for 30 min, and then incubated with primary antibodies against LRRC8A (17155-1-AP, Proteintech, 1:50), NSUN2 (20543-1-AP, Proteintech, 1:100), Caspase-3 (9662S, CST, 1:100), Ki67 (AF0198, Affinity, 1:100) at 4°C overnight. On the next day, the slides were incubated with the biotinylated secondary antibody at room temperature for 45 min, and then subjected to the DAB kit (ZSGB-BIO, ZLI-9018). The sections were stained with hematoxylin, decolorized with hydrochloric acid and ethanol, dehydrated and then mounted. The comprehensive score of immunostaining was evaluated independently by two professional pathologists.

### Cell proliferation and cell growth analyses

For cell proliferation assay, 1500 HeLa or 2500 SiHa cells were planted in 96-well plates with 200 μl fresh medium. Cell viabilities were measured after adding Cell Counting Kit-8 (CCK-8, Dojindo, Japan) for 2 hours. The stably transfected cells were plated into 96-well plates and incubated at 37°C for 6 hours until adherence and were referred to as 0 hour. Cell numbers were counted on 24, 48, 72, 96 hours. For the colony formation assay, transfected cells were seeded into 6-well plates. HeLa cells were plated with 1500 cells per well, and SiHa cells were plated with 2500 cells per well with 2 ml fresh medium and grown for 2 weeks. The colonies were fixed with 4% paraformaldehyde. After stained with crystal violet, the visible colonies were counted.

### Apoptosis assay

HeLa or SiHa cells were incubated with Annexin V and PI (DOJINDO, Japan) according to the instructions given by the manufacturer. The stained cells were subjected to flow cytometry. BD Accuri C6 flow cytometer (BD Biosciences, USA) was used to analyze apoptosis and the percentage of Annexin V+ PI- and Annexin V+ PI+ cells was calculated as the apoptosis rate.

### Cell migration and invasion assays

Migration and invasion assays were performed by using transwell chambers (8 μm, Corning Falcon). Stably transfected cells (HeLa, 6 × 10^4^; SiHa, 8 × 10^4^) and 250 μl serum-free medium were all added into the upper chamber of a transwell coated with or without Matrigel (Corning Incorporated, USA). The lower chamber was added with 650 μl cell culture medium containing 10% FBS. After incubation for 24 hours, the chambers were collected and stained by 0.5% crystal violet. The cells passing through the membrane were counted in three random fields.

### Cell swelling assays

For observing single cell morphological changes, stably transfected cells were planted in 12-well plates with 1 ml fresh medium for 6 hours until adherence. Then medium was changed into ddH_2_O and were referred to as 0 min. Morphological changes were assessed using an optical microscope. For cells that survived after swelling, 8000 HeLa and SiHa cells were treated in ddH_2_O for 0, 5, 10 min/0, 10, 20 min. Then cells were planted in 96-well plates with 200 μl fresh medium. After cells adherence, cell viabilities were measured after incubation with CCK-8 for 2 hours.

### Xenograft model in nude mice

Stably transfected HeLa cells (2 × 10^7^ cells) were injected subcutaneously into the thigh of BALB/c nude mice (4-6 weeks old, 5 mice/group). The length and width of the subcutaneous tumor was measured every 3 or 4 days. After injection for 22 or 24 days, the mice were euthanized, and the tumor weight was measured. The tumors were isolated and fixed in 4% formalin for IHC analysis. Animal studies were approved by the Ethics Committee of the Third Affiliated Hospital of Chongqing Medical University (2022033).

### RNA m^5^C dot blot

mRNA was purified by mRNA Isolation System IV kit (Poly Tract, USA). The m^5^C dot blot was performed on Amersham Hybond-N+ membrane (GE Healthcare, UK). After cross-linked by ultraviolet and blocked by PBST+5% milk for 1 hour, the membranes were incubated with the primary antibody against m^5^C (ab214727, Abcam, 1 µg/ml) overnight at 4°C. On the next day, the membrane was washed 3 times with PBST, and incubated with the secondary anti-rabbit antibody for 1 hour. Then the membrane was washed again with PBST. Finally, we used the chemiluminescence system to visualize the membrane. RNA levels were normalized with methylene blue staining to ensure consistency.

### RNA-sequencing (RNA-seq)

Total RNA was isolated using TRIzol Reagent (Sigma, USA). cDNA libraries were constructed by using NEBNext Ultra RNA Library Prep Kit for Illumina (New England BioLabs). All samples were sequenced on illumina Nova platform. Using STAR version 2.7.8a, all the clean reads were aligned to homo sapiens GENCODE GRCh38.v35 and counted the reads by featureCounts version 2.0.3. R package DESeq2 was used for differential expressed genes identification.

### RNA immunoprecipitation (RIP) assay

About 2 × 10^7^ HeLa cells or 4 × 10^7^ SiHa were lysed in RIP lysis buffer (HEPES 20 mM, 150 mM NaCl, 10 mM KCl, 5 mM EDTA, 5 mM MgCl_2_, 0.5% NP40, 10% glycerol). After incubation for 30 minutes on the ice, the lysate was harvested by centrifugation at 12,000 rpm for 10 min. The specific antibody (3 μg) or control IgG (3 μg) was incubated with the supernatant lysate overnight at 4°C. Next day, Dynabeads Protein A beads (Invitrogen, USA) were added into the lysate and incubated at 4°C for 3 hours. After washed three times with RIP lysis buffer, the co-precipitated RNA was extracted with Trizol Reagent (Sigma, USA). RNA was isolated and RT-qPCR was performed as described previously.

### Methylated RNA immunoprecipitation (MeRIP) assay

Total RNA was extracted by Trizol reagent (Sigma, USA) and then treated with RNase-free DNase set. After immunoprecipitated with anti-m^5^C antibody (3 μg/ml), Dynabeads Protein A beads (Invitrogen, USA) were added and incubated at 4°C for 3 hours. After washes, RNA was isolated, and RT-qPCR was performed as described previously.

### RNA stability assay

To test RNA stability, transfected HeLa or SiHa cells were seeded into 12-well plates. 5 µg/ml of actinomycin D (AbMole, USA) was added into each well and incubated for 0, 2, 4, 6 hours. Total RNA was extracted at the indicated time, and mRNA level was analyzed by qRT-PCR.

### Statistical analysis

All continuous data were shown as the mean ± SD and the significance of differences was evaluated by two tailed t-tests between two groups. It is considered statistical significant when p < 0.05, and in all cases, the p values were remarked as follows: ***P < 0.001, **P < 0.01, *P < 0.05, but statistically insignificant when P > 0.05. The growth rates difference was determined by ANOVA with repeated measures analysis of variances. The correlation between LRRC8A and NSUN2 expression in cervical cancer was analyzed by Spearman rank correlation analysis. All statistical analyses were performed using Graphpad Prism 8.

## Results

### LRRC8A is highly expressed and acts as a poor prognostic factor in CC

**Figure 1:**
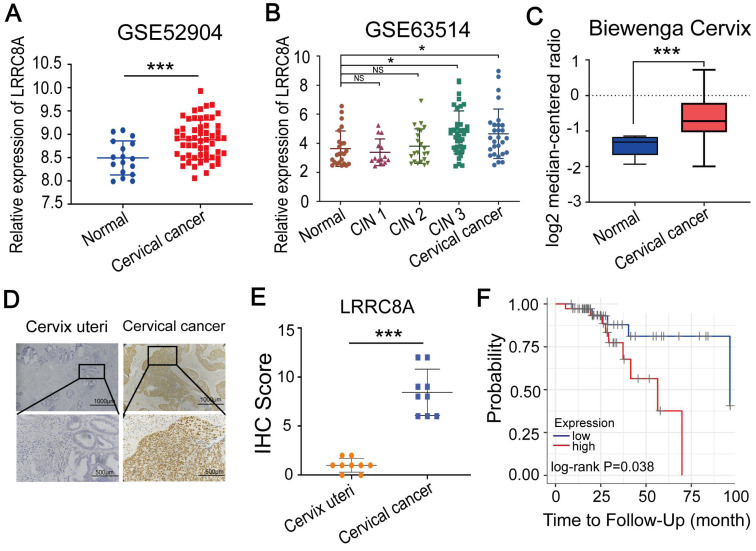
** Elevated expression of LRRC8A in CC predicts poor prognosis. (A, B)** Relative RNA levels of LRRC8A in CC tissues and normal cervical epithelium in GEO datasets. **(C)** Relative RNA levels of LRRC8A in CC tissues and cervix uteri in Oncomine database. **(D)** Representative IHC staining images of LRRC8A protein levels in CC tissues and cervical epithelium tissues. Scale bar, upper: 100 μm, lower: 500 μm. **(E)** The quantitative analysis of LRRC8A protein expression in CC tissues and cervical epithelium tissues assessed by IHC staining.** (F)** Kaplan-Meier analysis of CC patients' RFS based on LRRC8A expression by TIMER website. *P<0.05, **P < 0.01, ***P < 0.001, NS, not significant.

We firstly analyzed the mRNA levels of LRRC8 subunits (LRRC8A-E) 22-24 in CC tissues compared with normal tissues by using two GEO datasets (GSE63514 and GSE52904) and found that LRRC8A and LRRC8B were upregulated in CC tissues (Fig. 1A, B, Fig. S1A, B). We also observed the aberrantly overexpression of LRRC8A in CC in the Biewenga Cervix dataset according to the Oncomine database (Fig. 1C). Consistent with this, IHC assays showed that the protein abundance of LRRC8A was elevated in CC tumors when compared with normal cervix uteri tissues (Fig. 1D, E). Furthermore, we analyzed the survivals of CC patients with distinct expression of LRRC8 subunits by Kaplan-Meier survival analysis 25,26. Patients with higher LRRC8A expression had shorter recurrence free survival (RFS) time (Fig. 1F). However, the RNA levels of LRRC8B-E were not associated with of patients' RFS (Fig. S1C). Taken together, these data indicate that LRRC8A is frequently upregulated in CC and predicts poor prognosis for CC patients.

### LRRC8A promotes tumorigenesis of CC *in vitro* and *in vivo*

**Figure 2:**
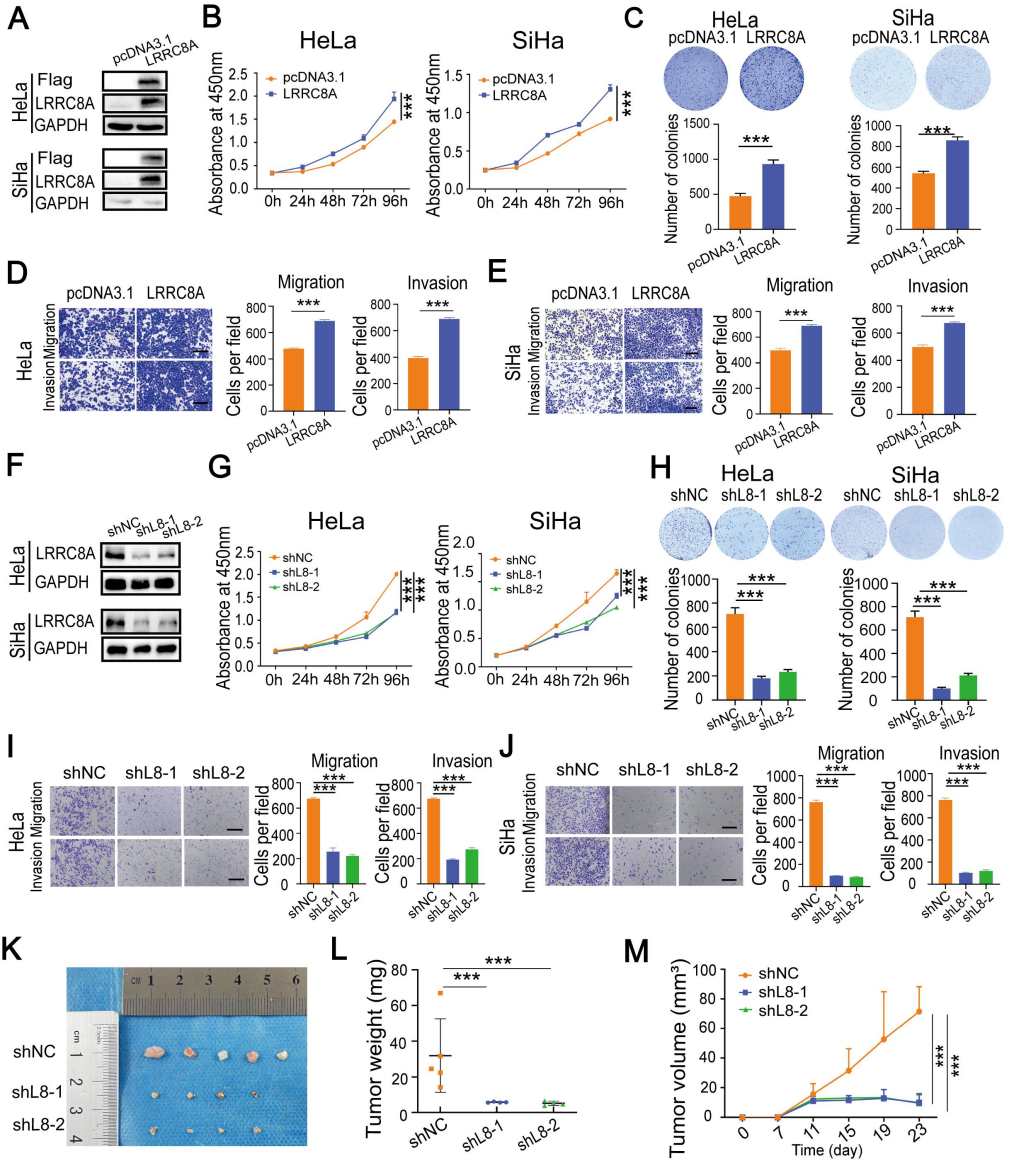
** LRRC8A promotes tumorigenesis of CC *in vitro* and *in vivo*. (A)** Western blot analysis of LRRC8A protein expression in CC cells with LRRC8A overexpression. **(B)** Cell growth curves of CC cells with or without LRRC8A overexpression by CCK-8 assays. **(C)** LRRC8A contributes to the proliferation of HeLa and SiHa cells as measured by colony formation assays. **(D, E)** Cell migration and invasion capacities of CC cells with LRRC8A overexpression. Scale bar, 200 μm. **(F)** LRRC8A knockdown was confirmed in HeLa and SiHa cells by western blot. **(G)** Effects of LRRC8A knockdown on abilities of CC cells' proliferation. **(H)** Colony formation assays were performed in CC cells upon LRRC8A knockdown. **(I, J)** Transwell assays detecting migration and invasion of CC cells with LRRC8A depletion. Scale bar, 200 μm. (K-M) Effects of LRRC8A knockdown on tumor weight and volume in the subcutaneous xenograft nude mouse model. Data are presented as means ± S.D: ***P < 0.001.

To explore the role of LRRC8A in CC cells, LRRC8A was overexpressed in HeLa and SiHa cells, and its effect on cell growth, migration and invasion was detected (Fig. 2A). CCK-8 and colony formation assays showed that LRRC8A overexpression resulted in enhanced cell growth. Transwell assays demonstrated that LRRC8A overexpression significantly increased the migration and invasion capabilities of CC cells (Fig. 2B-E). To further confirm the oncogenic effect of LRRC8A in CC cells, we constructed lentivirus-mediated LRCC8A knockdown HeLa cells and SiHa cells (Fig. 2F). As expected, reduced LRRC8A expression inhibited the proliferation, migration, and invasion of HeLa and SiHa cells (Fig. 2G-J). Moreover, subcutaneous tumorigenesis model was applied to assess the function of LRCC8A in CC *in vivo*. Compared with control group, xenograft tumors obtained from mice which burdened cells with LRRC8A knockdown were smaller and lighter (Fig. 2K-M, Fig. S2A). Together, these results demonstrate that LRCC8A facilitates tumorigenesis of CC *in vitro* and *in vivo*.

### LRRC8A keeps cellular homeostasis in CC cells

**Figure 3:**
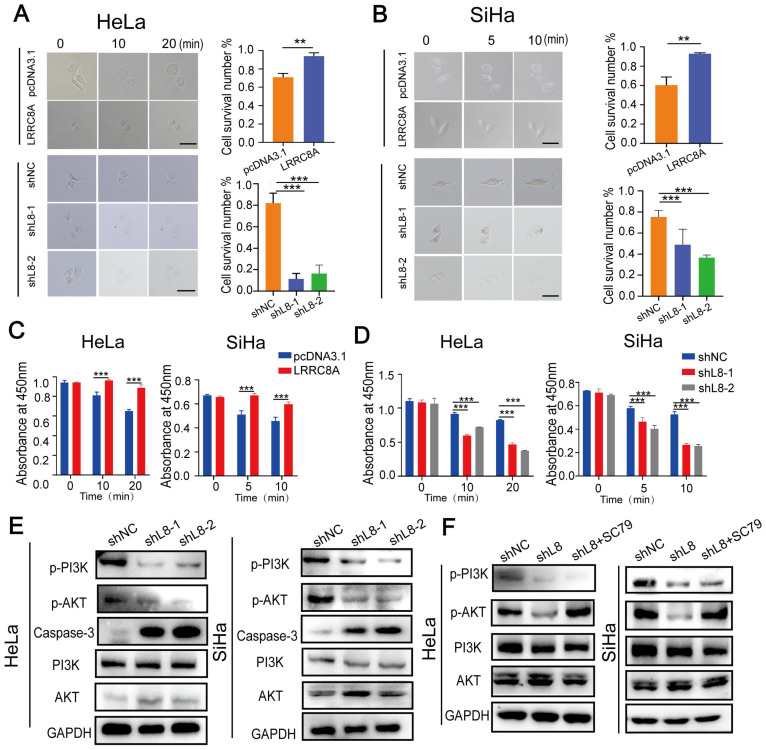
** LRRC8A maintains cellular homeostasis of CC cells under the hypotonic condition. (A, B)** Swelling analysis of CC cells with LRRC8A overexpression or knockdown under the the hypotonic condition. Scale bar, 10 μm. **(C, D)** CCK-8 assays detecting the survivals of CC cells with LRRC8A overexpression or knockdown upon the hypotonic treatment. **(E)** Changes of protein levels of p-PI3K p-AKT, PI3K, AKT and Caspase-3 in HeLa and SiHa cells upon LRRC8A knockdown. **(F)** Protein levels of p-PI3K, p-AKT, PI3K, AKT and in LRRC8A-depleted CC cells with or without SC79 treatment. Data are presented as means ± S.D: **P < 0.01, ***P < 0.001.

LRRC8A is an essential component of VRAC, a complex which is indispensable for homeostasis by resisting cell swelling. Cell size and cell deformability represent two key biophysical attributes of cells that have been demonstrated to modulate cancer invasion and metastasis 27. Thus, we detected whether LRRC8A was involved in maintaining cellular homeostasis in CC cells under hypotonic conditions. Under the optical microscope, we observed that LRCC8A-overexpressed cells remained constant both in volume and cell morphology compared with the control CC cells with slightly lagger gradually under hypotonic conditions. Conversely, LRCC8A knockdown accelerated swelling and broken of CC cells upon hypotonic solution treatment (Fig. 3A, B). We also measured the cell viability after treatment with hypotonic solution at different time points by CCK-8 assays. Results showed that compared with control group, LRRC8A overexpression promoted the cell survival after hypotonic solution treatment, but LRRC8A depletion significantly decreased the cell survival (Fig. 3C, D). Consistently, Annexin V/DAPI staining showed that depletion of LRRC8A results in increased apoptosis of CC cells, demonstrated that LRRC8A might promote survivals of CC cells through inhibiting apoptosis (Fig. S2B, C). Moreover, chemotherapy sensitivity assays revealed that CC cells with LRRC8A knockdown had higher sensitivity to cisplatin (Fig. S2D). These results suggested that LRRC8A might confer chemotherapy resistance in CC. PI3K/AKT, the essential signaling pathway associated with cell apoptosis and proliferation, has been reported regulated by LRRC8A 11,28,29,33. LRRC8A knockdown could reduce reactive oxygen species generation, resulting in the inactivation of PI3K/AKT signaling 30. LRRC8A-driven AKT phosphorylation can be inhibited by LY294002 (a PI3K phosphorylation inhibitor) 33. Consequently, we explored the effect of LRRC8A on the PI3K-AKT signaling pathway in CC cells, and the outcomes showed that LRRC8A knockdown inhibited PI3K-AKT signaling activation and promoted the expression of Caspase-3 in CC cells (Fig. 3E). In addition, the intervention of SC79 (an AKT phosphorylation agonist) could significantly rescue the suppression of AKT phosphorylation by LRRC8A knockdown (Fig. 3F). Furthermore, SC79 treatment improved the growth rate of cells with LRRC8A knockdown (Fig. S2E, F). Transwell assays exhibited that activation of AKT signaling by SC79 rescued the cell mobility of CC cells with LRRC8A knockdown (Fig. S2G).

### LRRC8A upregulation by m^5^C RNA methyltransferase NSUN2 in CC

**Figure 4:**
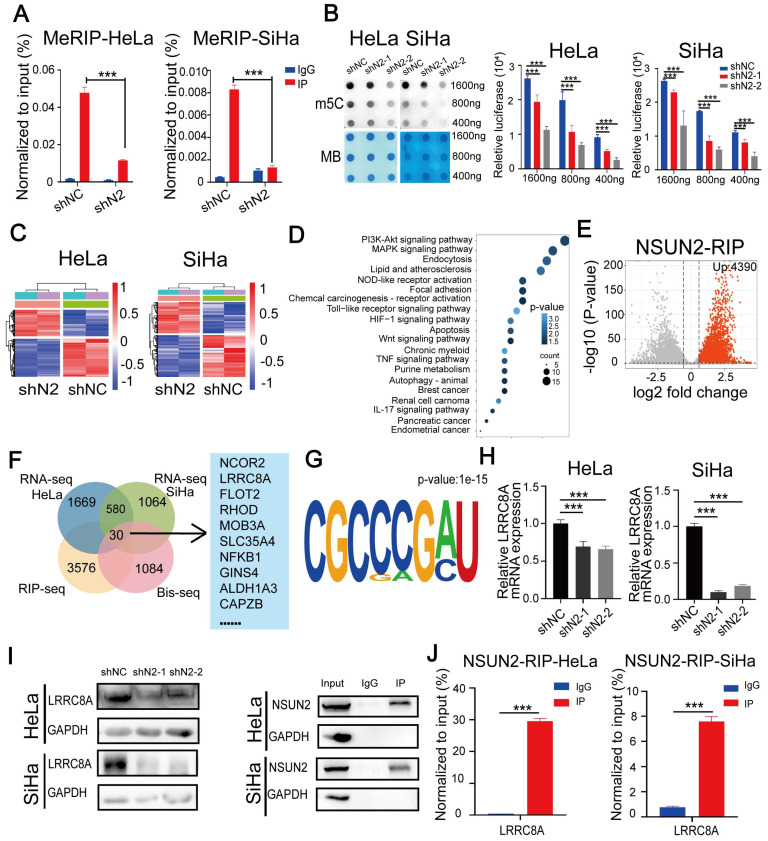
** NSUN2 promotes m^5^C modification and expression of LRRC8A mRNA. (A)** meRIP-PCR assays detecting the RNA m^5^C modification of LRRC8A in HeLa and SiHa cells upon NSUN2 knockdown. **(B)** Dot blot detecting the global m^5^C modification of mRNAs in CC cells with or without NSUN2 knockdown. Methylene blue (MB) staining was used as a loading control. **(C)** Heamap of differentially expressed genes from RNA-seq analysis of HeLa and SiHa cells upon NSUN2 knockdown. **(D)** Gene ontology analysis of differentially expressed genes described in (**C**). **(E)** Volcano plot of NSUN2 bound transcripts from RIP-seq analysis in SiHa cells. **(F)** A Venn diagram shows the potential targets of NSUN2 through the combined analysis of RNA-seq, RIP-seq from the present study and the reported RNA Bis-seq in HeLa cells. **(G)** Sequence frequency logo for the sequences proximal to mRNA m^5^C sites on the 30 NSUN2-bound mRNA. **(H, I)** The RNA and protein levels of LRRC8A in CC cells upon NSUN2 knockdown were measured. **(J)** RIP-PCR assays detecting the interaction between NSUN2 and LRRC8A mRNAs in HeLa and SiHa cells. IgG was used as an internal control. GAPDH was used as the negative control in western blot assays. Data are presented as means ± S.D: ***P < 0.001.

A recent RNA-BisSeq analysis described that LRRC8A might be regulated by m^5^C modification 31. To explore the relationship between LRRC8A and m^5^C modification, we performed meRIP-PCR by using an antibody specific to m^5^C within RNAs in CC cells. Results showed that LRRC8A was subjected to m^5^C modification and knockdown of the m^5^C methyltransferase NSUN2 could decrease the m^5^C modification of LRRC8A mRNA (Fig. 4A). Consistently, dot blot assays showed that NSUN2 knockdown decreased the global m^5^C level of mRNAs in both CC cells (Fig. 4B). Then we conducted RNA-seq upon NSUN2 knockdown and RIP-seq of NSUN2 to further study m5C-mediated gene regulation in CC cells. 1669 downregulated and 1251 upregulated genes in HeLa cells, and 1064 downregulated and 734 upregulated genes in SiHa cells were identified upon NSUN2 knockdown (Fig. 4C, Fig. S3A, B). Using gene ontology analysis, pathways enriched by these altered genes were essential for tumorigenesis such as PI3K-Akt signaling pathway (Fig. 4D). Meanwhile, NSUN2 bound 4390 transcripts by RIP-seq of NSUN2 in SiHa cells (Fig. 4E). Through overlapping these omics data, we found 30 transcripts, including LRRC8A, were m^5^C modified, bound by NSUN2 and differentially expressed after NSUN2 knockdown in both HeLa and SiHa cells (Fig. 4F). Furthermore, the common motifs including the m^5^C motif was found in these 30 transcripts targeted by NSUN2 (Fig. 4G), which was consistent with the previous study 31. We further confirmed LRRC8A regulation by NUSN2 by qRT-PCR and western blot assays. As expected, NSUN2 inhibition remarkably downregulated the RNA and protein levels of LRRC8A in CC cells (Fig. 4H, I). Results of RIP-PCR in HeLa and SiHa cells also revealed that NSUN2 bound to LRRC8A mRNA (Fig. 4J). Taken together, these data indicate that LRRC8A is upregulated by the m^5^C methyltransferase NSUN2.

### NSUN2 enhances tumorigenesis of CC

**Figure 5:**
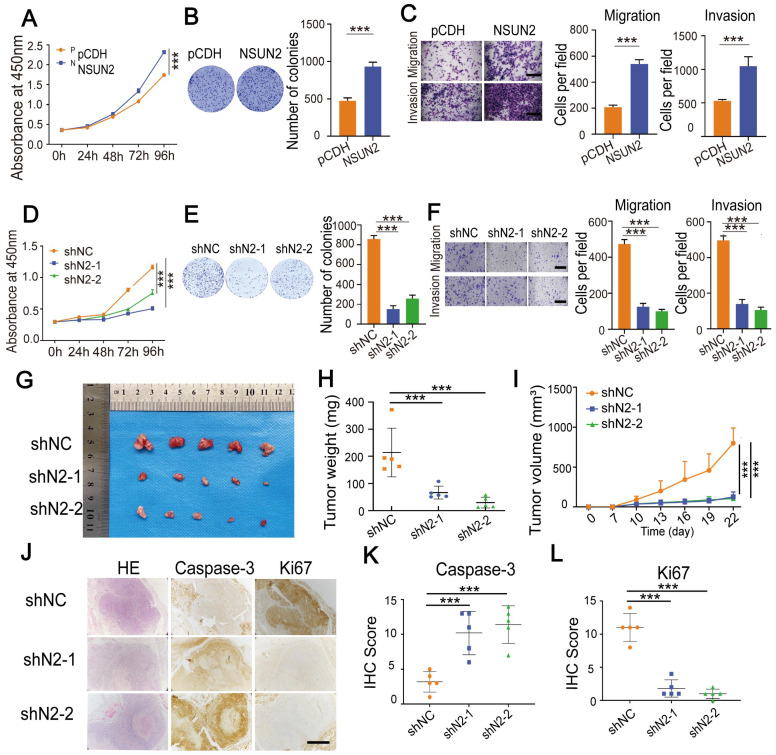
** NSUN2 exerts a tumor-promoting role in CC**. **(A)** CCK-8 assays showed the growth of HeLa cells upon NSUN2 overexpression. **(B)** The effect of NSUN2 overexpression on the colony formation of HeLa cells. **(C)** Transwell cell migration and invasion analysis of NSUN2 overexpressed and control HeLa cells. Scale bar, 200 μm. **(D)** The effect of NSUN2 knockdown on cell growth was determined by CCK-8 assays. **(E)** Colony formation assays were performed in NSUN2 knockdown and control cells. **(F)** Migration and invasion assays of HeLa cells with or without NSUN2 knockdown. Scale bar, 200 μm. **(G-I)** Effects of NSUN2 knockdown on tumor weight and volume in the subcutaneous xenograft nude mouse model. **(J-L)** Representive IHC staining images and quantative analysis of Ki67 and Caspase-3 in xenograft tumors from NSUN2 knockdown and control cells treated nude mice. Scale bar, 500 μm. Statistical analyses showed the IHC staining of Ki67 and Caspase-3 from xenograft tumors. Data are presented as means ± S.D: ***P < 0.001.

Next, we investigated the role of NSUN2 in CC cells. Overexpressing NSUN2 notably promoted cell proliferation, migration and invasion (Fig. 5A-C, Fig. S4A-D). In contrast, knocking down NSUN2 substantially suppressed cell proliferation, migration, and invasion of CC cells (Fig. 5D-F, Fig. S4E-G). To explore the oncogenic role of NSUN2 *in vivo*, we conducted subcutaneous tumor formation experiments. Knockdown of NSUN2 resulted in the smaller and lighter xenograft tumors that formed in nude mice when compared with the control group (Figure 5G-I, Fig. S4H). IHC assays showed that tumors formed by cells with NUSN2 knockdown exhibited lower Ki67 but higher Caspase-3 levels (Fig. 5J-L). What's more, the mRNA level of NSUN2 in CC tissues compared with normal tissues was significantly up-regulated (Fig. S4I-J). IHC assays showed that the protein abundance of NSUN2 was elevated in CC tumors compared with normal cervix uteri tissues (Fig. S4K-L). The survivals analysis showed that patients with higher LRRC8A expression had shorter recurrence free survival (RFS) time (Fig. S4M). Thus, these results reveal that NSUN2 enhances tumorigenesis of CC and associated with poor prognosis.

### m^5^C modification stabilizes LRRC8A mRNA in CC

**Figure 6:**
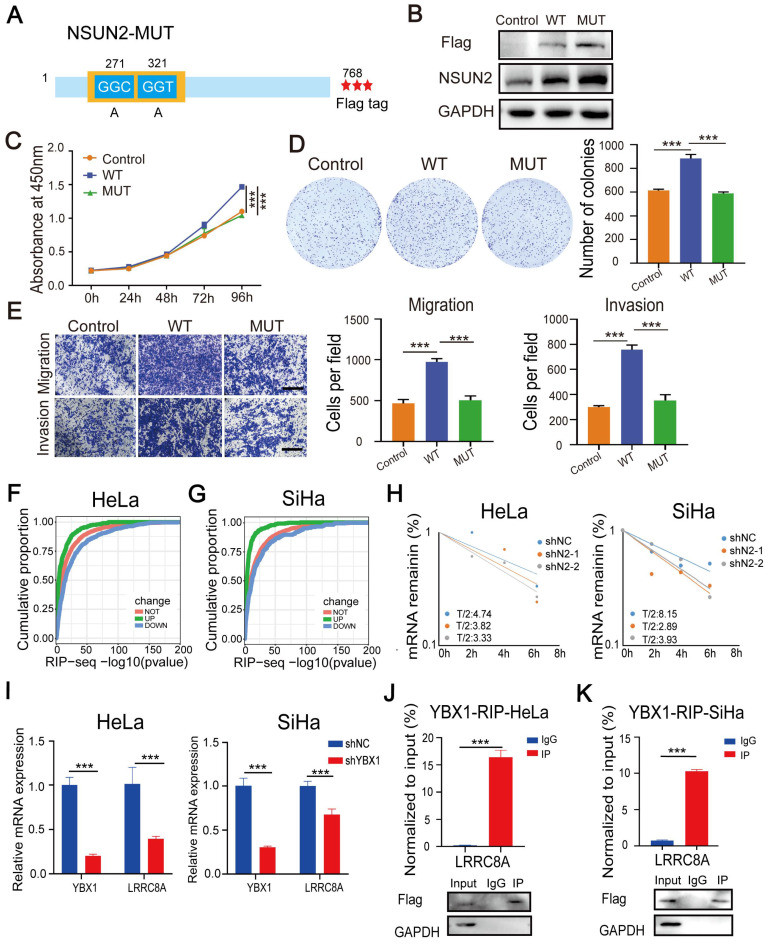
** NSUN2 facilitates tumorigenesis of CC cells in an m5C-dependent manner. (A)** Schematic of the NSUN2 mutant (NSUN2-MUT). **(B)** Western blot confirmed the overexpression of wild-type or mutated NSUN2 in CC cells. **(C)** CCK-8 assays of SiHa cells with forced expression of wild-type or mutated NSUN2. **(D)** Colony formation assays were performed in SiHa cells with forced expression of wild-type or mutated NSUN2. **(E)** Transwell cell migration and invasion assays of SiHa cells with NSUN2-WT and NSUN2-MUT. Scale bar, 200 μm. **(F, G)** Cumulative proportion of NSUN2 bound transcripts with differentially expressed genes from RNA-seq analysis of NSUN2 knockdown in HeLa and SiHa cells. **(H)** Effects of NSUN2 knockdown on mRNA half-life of LRRC8A by RNA stability assays. **(J, K)** RT-qPCR detecting RNA levels of LRRC8A in HeLa and SiHa cells upon YBX1 knockdown. **(I)** RIP-PCR assays detecting the interactions between YBX1 and LRRC8A mRNA in HeLa and SiHa cells. IgG was used as an internal control. GAPDH was used as the negative control in western blot assays. Data are presented as means ± S.D: ***P < 0.001.

To further explore the tumor-promoting role of NSUN2 dependently on m5C modification in CC, we constructed a wide-type NSUN2 expression plasmid (NSUN2-WT) and an m^5^C methyltransferase-mutated plasmid (NSUN2-MUT) by introducing point mutations in releasing and catalytic sites (cysteine 271 and 321) (Fig. 6A). Then, CC cells were transfected with wild-type or mutated NSUN2 (Fig. 6B). NSUN2-WT substantially promoted cell proliferation, migration and invasion. However, overexpressing NSUN2 mutant had no obvious effects on the malignant phenotype of SiHa cells (Fig. 6C-E). The exactly alike conclusion was also validated in the HeLa cells (Fig. S5A-D). Further RIP assays showed that the NSUN2 mutation accounted for the dramatically reduced level of LRRC8A mRNA bound by NSUN2 in CC cells (Fig. S5E). NSUN2-mediated m^5^C modification was reported to regulate RNA stability 16,17, and the results of RNA-seq and RIP-seq also showed that transcripts bound by NSUN2 were more likely to be downregulated in the RNA level upon NSUN2 depletion (Fig. 6F, G). In this way, we detected the effect of NSUN2 on the half-life of LRRC8A mRNA. The results showed that knockdown of NSUN2 shortened the half-life of LRRC8A mRNA (Fig. 6H). Considering that the RNA binding protein YBX1 was involved in regulating the stability of transcripts with m^5^C modification, we investigated the influence of YBX1 on LRRC8A expression. The RT-qPCR results showed that YBX1 knockdown led to lower mRNA level of LRRC8A (Fig. 6I). RIP assays by using the antibody specific to YBX1 also showed that YBX1 accumulated LRRC8A mRNA significantly in both HeLa and SiHa cells (Fig. 6J, I). Taken together, these results indicate that NSUN2-mediated m^5^C modification stabilizes LRRC8A mRNA.

### NSUN2-LRRC8A axis is crucial for malignant growth and metastasis of CC cells

**Figure 7:**
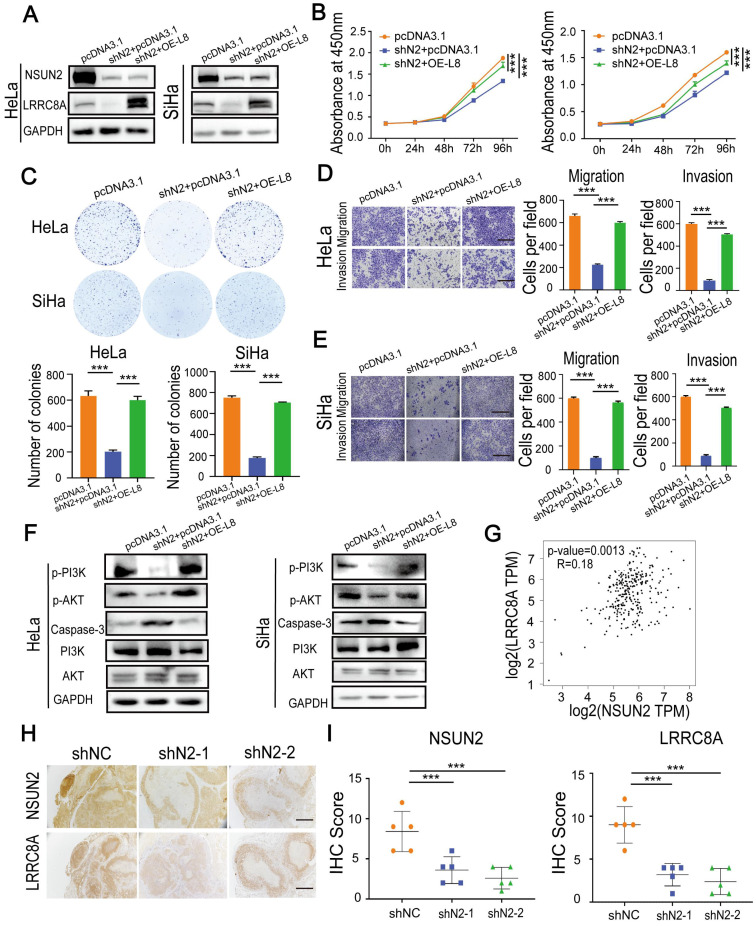
** Overexpression of LRRC8A rescues the phenotype of CC cells with NSUN2 knockdown. (A)** Western blot confirmed the protein expression of NSUN2 and LRRC8A in NSUN2-depleted CC cells with or without LRRC8A re-expression. **(B)** Overexpression of LRRC8CA rescues the cell proliferation suppressed by NSUN2 knockdown in HeLa and SiHa cells by CCK-8 assays. **(C)** Overexpression of LRRC8A restores the colony formation of CC cells suppressed by NSNU2 knockdown. **(D, E)** Cell migration and invasion analysis of NSUN2-depleted CC cells with or without LRRC8A re-expression. Scale bar, 200 μm. **(F)** Protein expression levels of p-PI3K, p-AKT, PI3K, AKT and Caspase-3 in NSUN2-depleted CC cells with or without LRRC8A re-expression. **(G)** Spearman's correlation analysis between mRNA levels of NSUN2 and LRRC8A in cervical cancer. **(H)** Representative images showing protein levels of NSUN2 and LRRC8A in nude mouse xenograft tumors. Scale bar, 500 μm. **(I)** Statistical analysis of IHC score showing NSUN2 and LRR8CA levels in xenograft tumors. Data are presented as means ± S.D: ***P < 0.001.

To further confirm the synergetic carcinogenic effect of LRRC8A with NSUN2 in CC, LRRC8A was re-expressed in NSUN2 knockdown cells (Fig. 7A), and the effects on cell proliferation, migration and invasion were analyzed. Ectopic expression of LRRC8A largely rescued the inhibition of proliferation rate caused by NSUN2 knockdown (Fig. 7B, C). The suppressive effect of NUSN2 knockdown on migration and invasion capacities of SiHa and HeLa cells was also partially reversed by LRRC8A restoration (Fig. 7D, E). Moreover, p-PI3K and p-AKT expression was significantly decreased with induced Caspase-3 protein level in NSUN2-depleted CC cells, and overexpressing LRRC8A could rescue PI3K-AKT signaling and inhibit Caspase-3 expression in CC cells (Fig. 7F, Fig. S6A). In addition, the correlation analysis suggested that there was a positive correlation between the mRNA level of NSUN2 and LRRC8A in CC (Fig. 7G). In agreement with this, IHC staining exhibited that the protein expression level of and LRRC8A was remarkably declined in tumors formed by NSUN2-depleted Hela cells (Fig. 7H, I). Together, these results indicate that the NSUN2-m^5^C-LRRC8A axis is crucial for cervical tumorigenesis.

## Discussion

LRRC8A was identified as an essential component of VRAC which plays a pivotal role in cell volume regulation 9. It was reported that LRRC8A regulates B- and T-cell development, chemoresistance, apoptosis, small molecule transportation 12,13,32-35. However, limited information is currently available on the role of LRRC8A in cancer. In this study, we found that LRRC8A expression was significantly upregulated in CC and the high expression of LRRC8A was identified as a poor prognostic factor in CC. Through *in vitro* and *in vivo* experiments, we confirmed that LRRC8A depletion significantly inhibited the proliferation, migration and invasion of CC cells and promoted apoptosis.

Our results demonstrated that LRRC8A promotes tumorigenesis in CC. Consistently, LRRRC8A was demonstrated to be related with growth and metastasis of hepatocellular carcinoma and gastric cancer and prognosis in colon cancer 12,13,36. Extensive research has confirmed that cellular homeostasis regulated by various ion transporters plays a crucial role in cancers. Zinc transporters mediated signaling pathways and zinc levels lead to cancer cell proliferation and metastasis 41. Zinc transporter SLC39A13/ZIP13 knockout depresses the malignant phenotypes of ovarian cancer cells both *in vitro* and *in vivo* 42. Increased expression of the copper efflux transporter ATP7A mediates resistance to cisplatin, carboplatin, and oxaliplatin in ovarian cancer cells 43. Na+, HCO_3_- cotransporter NBCn1 (Slc4a7) is increased in ErbB2-induced breast cancer tissue compared to normal, and disrupting NBCn1 expression delays ErbB2-induced breast cancer development 44. However, the mechanisms of LRRC8A upregulation as well as the pathways involved in its tumor-promoting role were unexplored.

RNA modification is an important post-transcriptional regulation. By analyzing RNA-BisSeq data of HeLa cells 31, we found that LRRC8A mRNA may have m^5^C sites. RNA m^5^C modification is a widespread post-transcriptional RNA modification and participates in various cellular and physiological processes. NSUN2 is the main enzyme catalyzing m^5^C formation and plays an important role in the regulation of RNA fate, such as RNA stability, RNA processing, RNA export, and mRNA translation 15-20. To explore the relationship among LRRC8A and m^5^C modification, we established RNA-seq in NSUN2 knockdown CC cells and found that LRRC8A was downregulated upon NSUN2 knockdown. Correlation analysis showed LRRC8A expression was positively correlated with NSUN2 expression in CC. m^5^C methyltransferase NSUN6 was also demonstrated to mediate m^5^C methylation on mRNA 45. However, no positive correlation between the mRNA levels of NSUN6 and LRRC8A in CC was observed (Fig. S6B), which is consistent with that only NSUN2 affects the global m^5^C level in mRNAs in HeLa cells 31. MeRIP assays showed that LRRC8A mRNA was modified with m^5^C and NSUN2 depletion could decrease the level of m^5^C modification on LRRC8A mRNA. Also, RIP-seq in SiHa cells as well as RIP-PCR assays both confirmed that NSUN2 could specifically interact with LRRC8A mRNA. YBX1 is an m^5^C reader protein, and we found that YBX1 knockdown led to lower mRNA level of LRRC8A in CC cells. Since m^5^C modification was reported to be associated with regulating the stability of mRNA 16,17, we treated cells with actinomycin D and uncovered that knockdown of NSUN2 shortened the half-life of LRRC8A mRNA. These results indicate that NSUN2 promotes LRRC8A mRNA stability through m^5^C deposition and upregulates its expression in CC.

Notably, gene ontology analysis showed that differentially expressed genes in NSUN2-loss cells significantly enriched PI3K-Akt signaling pathway. PI3K/AKT pathway plays vital roles in various cellular processes and is involved in many diseases such as malignant tumors 37. Several studies have reported that CC patients show abnormal activation of PI3K/AKT signaling, which may provide potential therapeutic targets of CC 38-40. In the present study, we demonstrated that knockdown expression of NSUN2 and LRRC8A both could depress PI3K/AKT signaling pathway activation. Nevertheless, how this signaling pathway was activated in CC by NSUN2 and LRRC8A require more detailed and systematic studies. Apart from PI3K/AKT pathway, differentially expressed genes in NSUN2-depleted CC cells are enriched in multiple other oncogenic pathways, and NSUN2-deplete CC cells revealed the reduced global m^5^C level of mRNA. Thus, we explored the role of NSUN2 in CC cells. Results demonstrated that NSUN2 played a tumor-promoting role in CC. Depletion of NSUN2 significantly inhibited cell growth and metastasis, whereas ectopic expression of LRRC8A in NSUN2-depleted cells could largely rescue the tumor-suppressive effect of NSUN2 knockdown. Considering that HPV infection is an important causative factor of CC, we test whether NSUN2-LRRC8A regulation was associated with HPV infection. Results showed that the mRNA levels of NSUN2 and LRRC8A had no statistical difference in HPV+ CC compared with HPV- CC tissues according to analyzed two GEO datasets (Fig. S6C). Interestingly, recent research unveiled that high expression of the N6-methyladenosine methyltransferase METTL3 is associated with HPV status and CC patients' poor prognosis 46. Thus, the relationship between RNA modifications including the m^5^C modification and HPV-infection needs more investigations.

**Figure 8:**
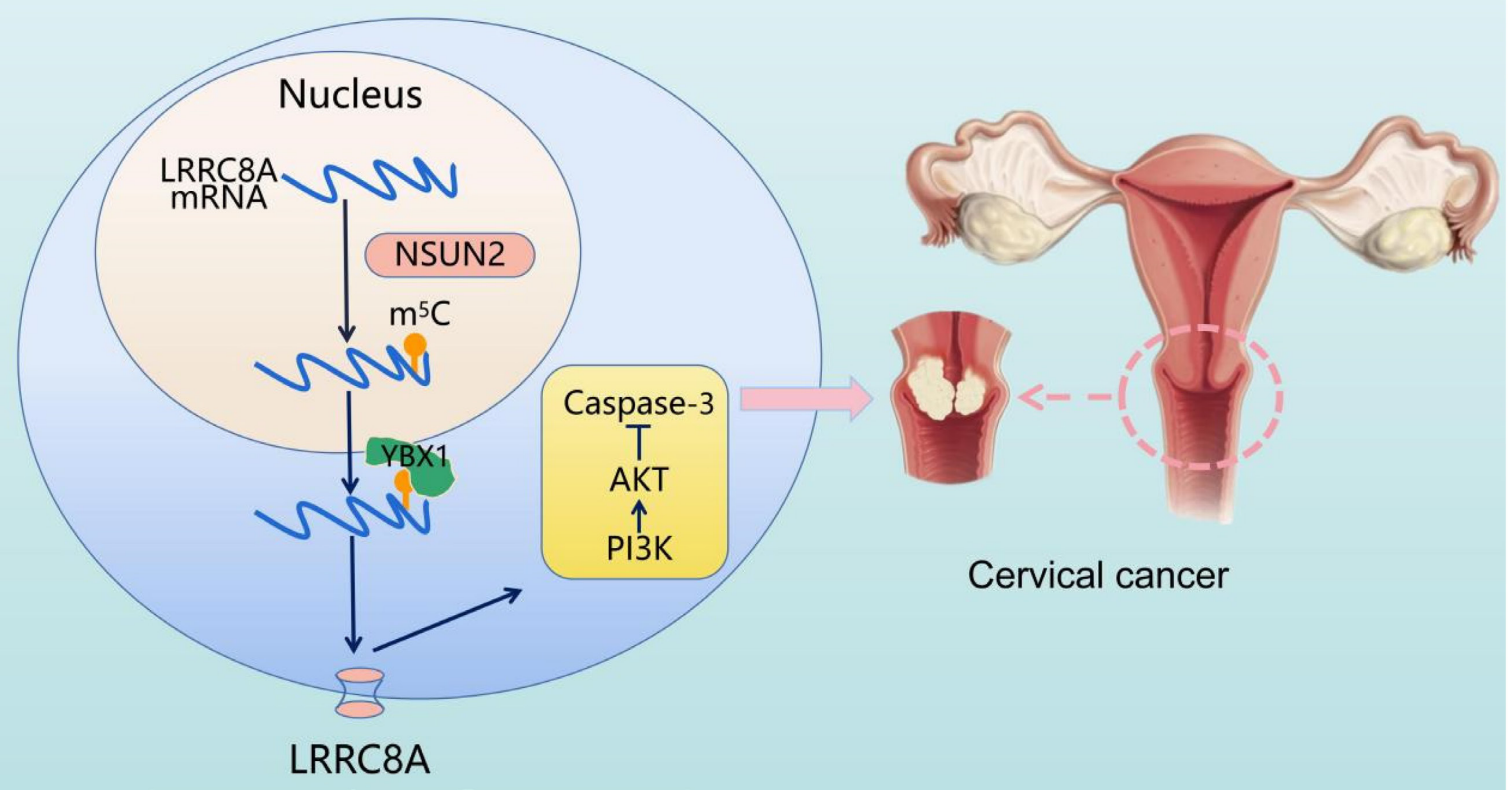
Schematic diagram illustrating the proposed mechanisms of m^5^C-mediated upregulation of LRRC8A in tumorigenesis of CC.

In conclusion, elevated expression of LRRC8A in cervical cancer predicts a poor prognosis and promotes tumorigenesis *in vitro* and *in vivo*. The m^5^C modification enhances LRRC8A mRNA stability and upregulates its expression followed by PI3K/AKT signaling pathway activation in CC (Fig. 8). These findings contribute to our understanding the role of the NSUN2-m^5^C-LRRC8A axis in tumorigenesis of CC, and it might be a potential target for CC treatment.

## Supplementary Material

Supplementary figures and table.
