# Supplementary material for: Upregulation of LRRC8A by m5C modification-mediated mRNA stability suppresses apoptosis and facilitates tumorigenesis in cervical cancer
Source: Int J Biol Sci. 2023 Jan 1;19(2):691–704. doi: 10.7150/ijbs.79205 (PMC9830503; doi:10.7150/ijbs.79205)
Supplement: Supplementary file 1 — Supplementary figures and table. [file ijbsv19p0691s1.pdf]

Supplementary figures and legend

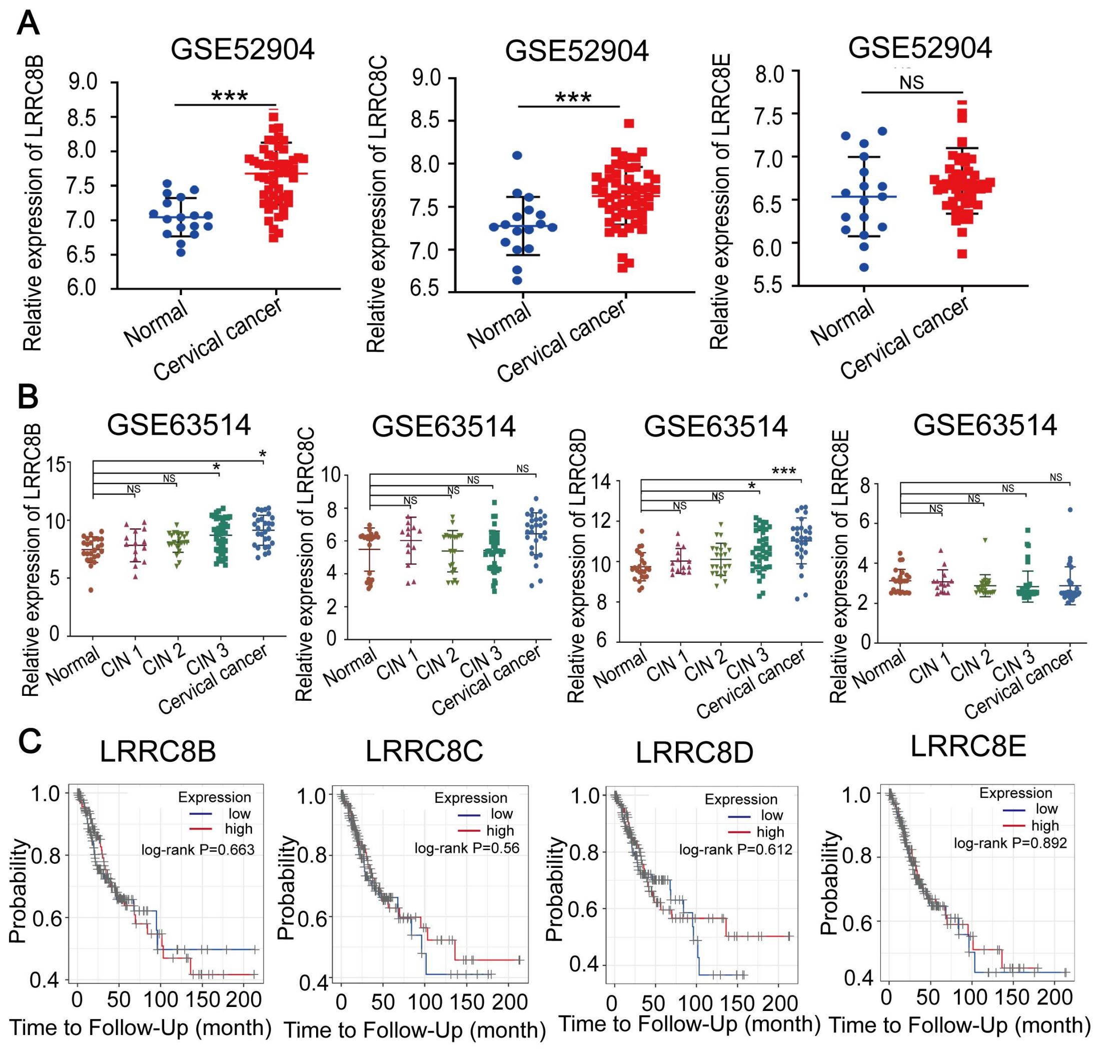

Fig S1. (A and B) Relative RNA levels of LRRC8B, LRRC8C and LRRC8E in CC tissues and normal cervical epithelium in GEO datasets (GSE52904 and GSE63514). **(C)** Kaplan-Meier analysis of CC patients' RFS based on LRRC8B, LRRC8C, LRRC8D, LRRC8E expression by TIMER website. *P<0.05, **P < 0.01, ***P < 0.001, NS, not significant.

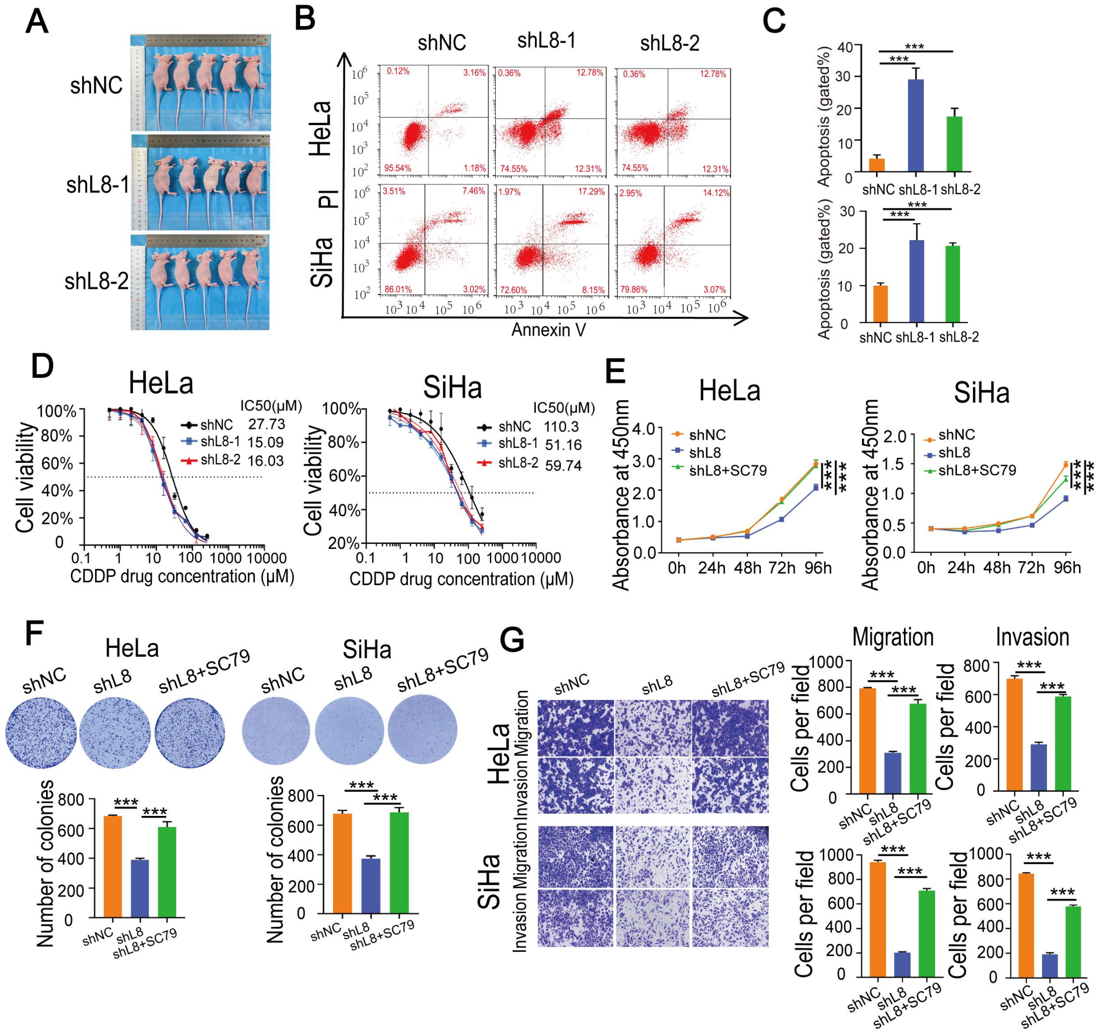

Fig S2. **(A)** Images of xenograft tumors derived from subcutaneous implantation of LRRC8A knockdown and control cells on nude mice. **(B)** The effect of LRRC8A knockdown on apoptosis of cervical cancer cells. **(C)** Statistical analysis percentage of early-and late-apoptotic cells in LRRC8A knockdown cells. **(D)** Cell viability of control and LRRC8A knockdown cells after treatment with increasing concentrations of cisplatin for 24 h. n = 3. **(E)** The AKT phosphorylation agonist rescues CC cells' proliferation suppressed by LRRC8A depletion. **(F)** SC79 treatment restores the colony formation of CC cells suppressed by LRRC8A knockdown. **(G)** Cell migration and invasion analysis of LRRC8A knockdown in HeLa and SiHa cells with or without SC79 treatment. Scale bar, 200 μm. Data are presented as means ± S.D: ***P < 0.001.

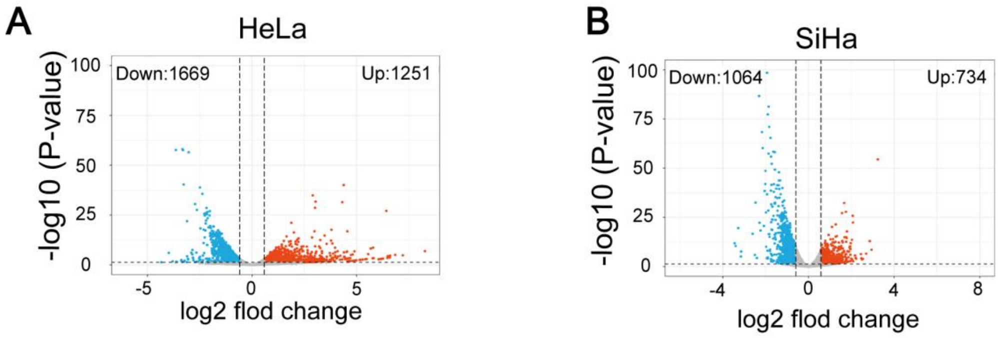

Fig S3. **(A)** Volcano plot of differentially expressed genes from RNA-seq analysis of NSUN2 knockdown in HeLa cells. **(B)** Volcano plot of differentially expressed genes from RNA-seq analysis of NSUN2 knockdown in SiHa cells.

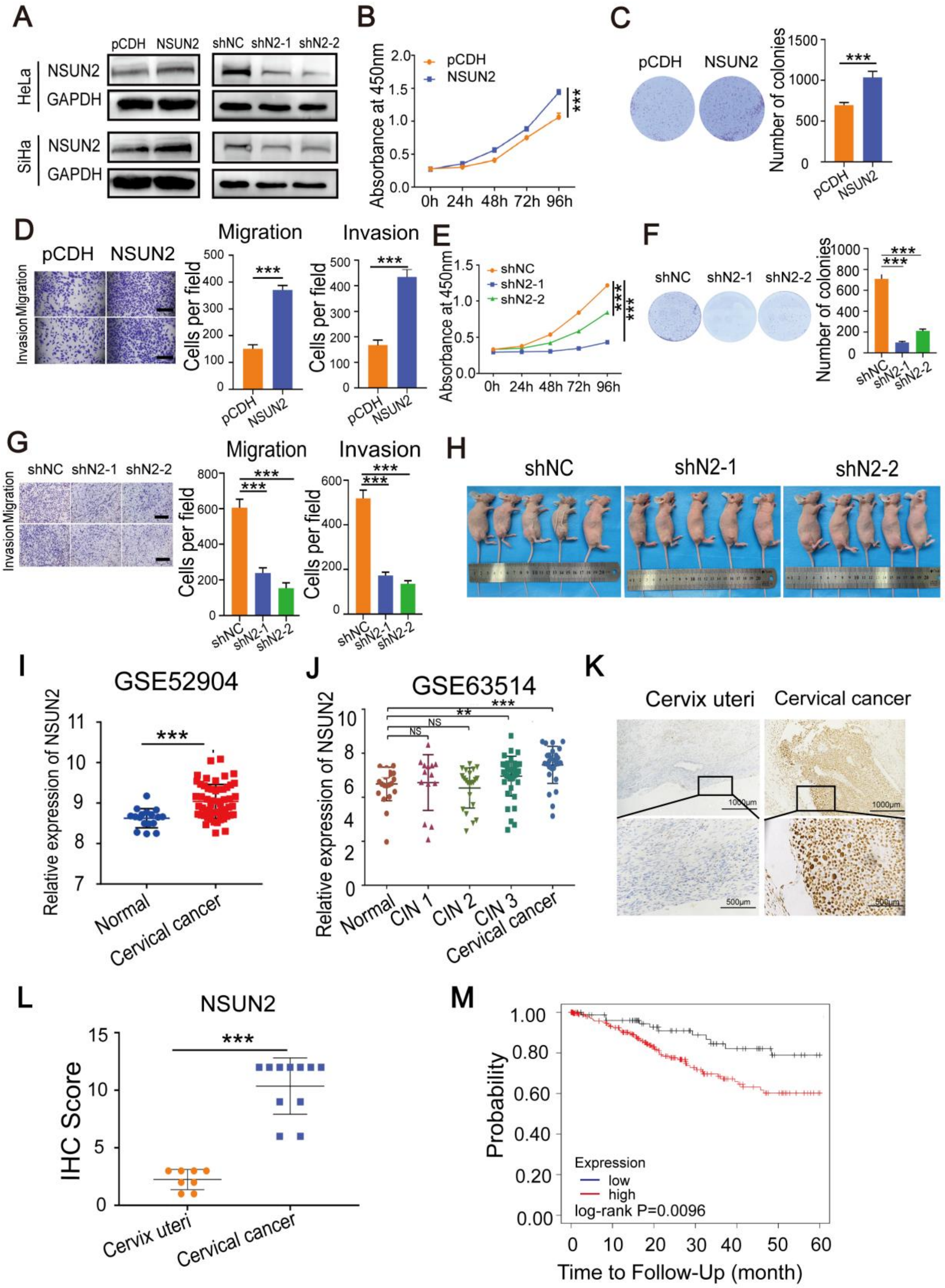

Fig S4. (A) Protein expression of NSUN2 overexpression and knockdown in HeLa and SiHa cells. **(B)** CCK-8 assays showed the proliferation of SiHa cells upon NSUN2 overexpression. **(C)** Cell colony formation of SiHa cells with NSUN2 overexpression. **(D)** Transwell cell migration and invasion analysis of NSUN2 overexpression and control cells. Scale bar, 200 μm. **(E)** The effect of NSUN2 knockdown on cell growth was determined by CCK-8 assays. **(F)**

Colony formation assays were performed in NSUN2 knockdown and control cells. **(G)** Migration and invasion analysis of SiHa cells upon NSUN2 knockdown. Scale bar, 200 μm. **(H)** Images of xenograft tumors derived from subcutaneous implantation of NSUN2 knockdown and control cells on nude mice. **(I and J)** Relative RNA levels of NSUN2 in CC tissues and normal cervical epithelium in GEO datasets (GSE52904 and GSE63514). **(K)** Representative IHC staining images of NSUN2 protein levels in CC tissues and cervical epithelium tissues. Scale bar, upper: 100 μm, lower: 500 μm. **(L)** The quantitative analysis of NSUN2 protein expression in CC tissues and cervical epithelium tissues assessed by IHC staining. **(M)** Kaplan-Meier analysis of CC patients' RFS based on NSUN2 expression. Data are presented as means ± S.D: ***P < 0.001.

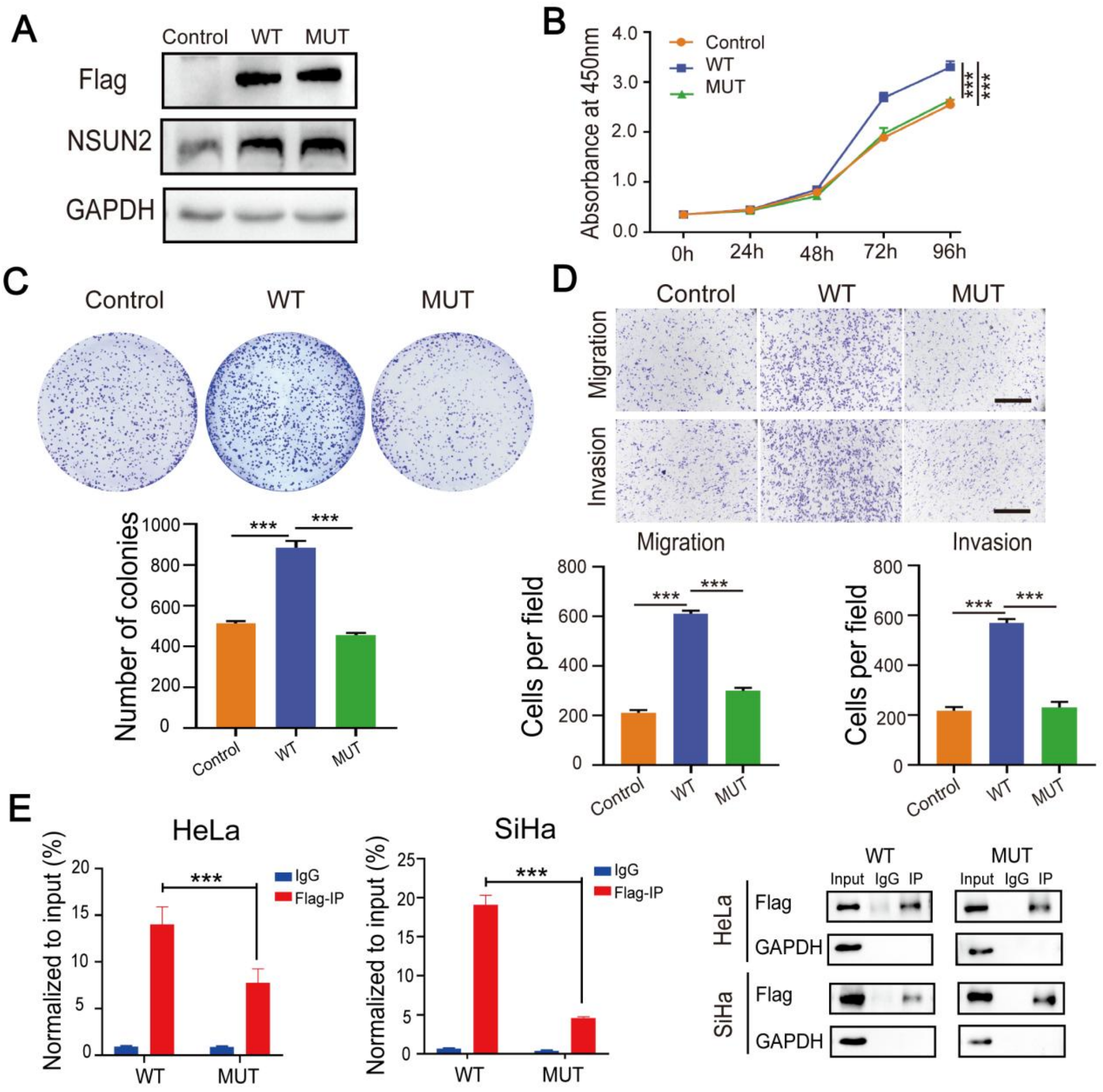

Fig S5. (A) Western blotting assays showed the protein levels of NSUN2 in NSUN2-WT, NSUN2-MUT and control HeLa cells. **(B)** CCK-8 assays of HeLa cells with forced expression of wild-type or catalytic-mutant NSUN2. **(C)** Colony formation assays were performed in CC cells with forced expression of wild-type or mutated NSUN2. **(D)** Transwell cell migration and invasion assay of HeLa cells with forced expression of wild-type or mutated NSUN2. Scale bar, 200 μm. **(E)** RIP-PCR assays detecting the interactions between wild-type or mutated NSUN2 and LRRC8A mRNA. IgG was used as an internal control. GAPDH was used as the negative control in western blot assays. Data are presented as means ± S.D: ***P < 0.001.

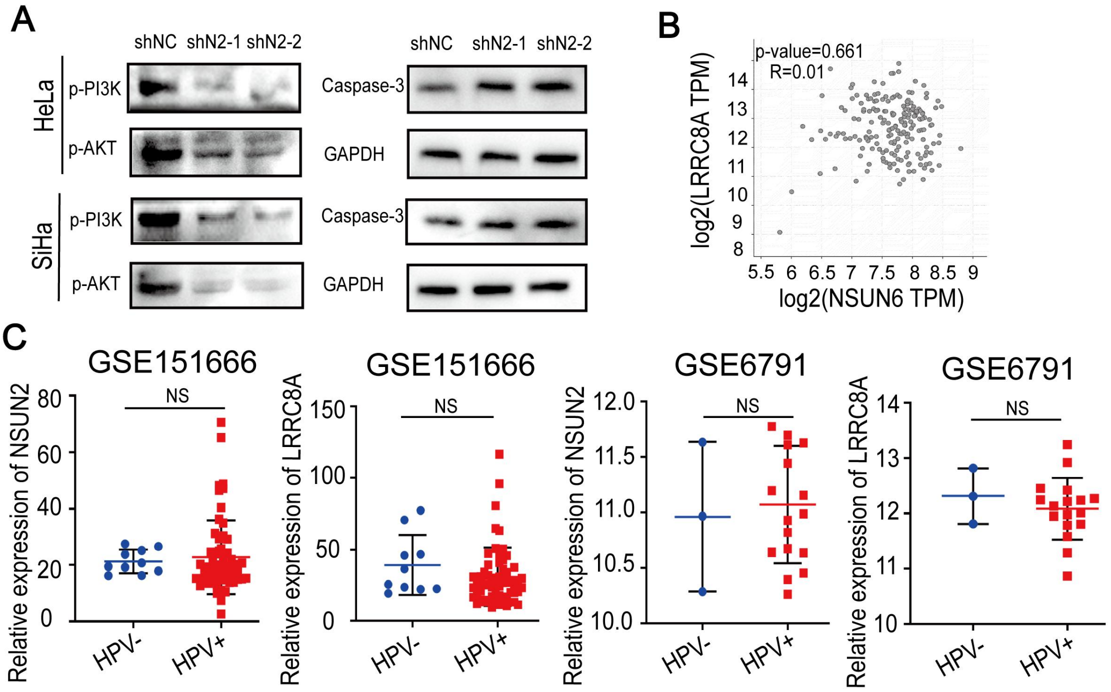

Fig S6. (A) Western blot assays showed protein levels of p-AKT, p-PI3K and Caspase-3 in HeLa and SiHa cells upon NSUN2 knockdown. **(B)** Spearman's correlation analysis between mRNA levels of NSUN6 and LRRC8A in cervical cancer. **(C)** Relative RNA levels of NSUN2 and LRRC8A in HPV negative and HPV positive CC tissues in GEO datasets. NS, not significant.

Supplementary table 1: Sequences of primers and oligos used in this study

	Forword	Reverse
NSUN2	CAAGCTGTTCGAGCACTA CTAC	CTCCCTGAGAGCGTCCAT GA
GAPDH	CTGGGCTACACTGAGCA CC	AAGTGGTCGTTGAGGGCA ATG
LRRC8A	TGTGTGCTATGAGAACCG ACT	GTGCGCGGGAATTTGAAC C
YBX1	GGGGACAAGAAGGTCAT CGC	CGAAGGTACTTCCTGGGG TTA
18S	CGATAACGAACGAGACTC TGGC	CGGACATCTAAGGGCATC ACA
shLRRC8A-1	CCGGCCGCAACAAGATC GAGAAGATCTC GAGATCTTCTCGATCTTG TTGCGGTTTTTG	AATTCAAAAACCGCAACAA GATCGAGAAG ATCTCGAGATCTTCTCGAT CTTGTTGCGG
shLRRC8A-2	CCGGCATCTGCTACACCG TCTACTACTCGAGTAGTA GACGGTGTAGCAGATGTT TTTG	AATTCAAAAACATCTGCTAC ACCGTCTACTACTCGAGTA GTAGACGGTGTAGCAGATG
shNSUN2-1	CCGGGCGTGTTAGAAATC ACTTGTTCTCGAGAACAA GTGATTTCTAACACGCTT TTTG	AATTCAAAAAGCGTGTTAG AAATCACTTGTTCTCGAGA ACAAGTGATTTCTAACACG C
shNSUN2-2	CCGGGCACACAAATTTAA GTCGAAACTCGAGTTTCG ACTTAAATTTGTGTGCTTT TTG	AATTCAAAAAGCACACAAA TTTAAGTCGAAACTCGAGT TTCGACTTAAATTTGTGTG C
shYBX1	CCGGCCAGTTCAAGGCA GTAAATATCTCGAGATAT TTACTGCCTTGAACTGGT TTTTG	AATTCAAAAACCAGTTCAA GGCAGTAAATATCTCGAGA TATTTACTGCCTTGAACTG G
